# Pheochromocytomas and Paragangliomas: Bypassing Cellular Respiration

**DOI:** 10.3390/cancers11050683

**Published:** 2019-05-16

**Authors:** Alberto Cascón, Laura Remacha, Bruna Calsina, Mercedes Robledo

**Affiliations:** 1 Hereditary Endocrine Cancer Group, Spanish National Cancer Research Centre (CNIO), 28029 Madrid, Spain; l.remacha.m@gmail.com (L.R.); bcalsina@cnio.es (B.C.); 2 Centro de Investigación Biomédica en Red de Enfermedades Raras (CIBERER), 28029 Madrid, Spain

**Keywords:** pheochromocytoma, paraganglioma, TCA cycle, germline mutation

## Abstract

Pheochromocytomas and paragangliomas (PPGL) are rare neuroendocrine tumors that show the highest heritability of all human neoplasms and represent a paradoxical example of genetic heterogeneity. Amongst the elevated number of genes involved in the hereditary predisposition to the disease (at least nineteen) there are eleven tricarboxylic acid (TCA) cycle-related genes, some of which are also involved in the development of congenital recessive neurological disorders and other cancers such as cutaneous and uterine leiomyomas, gastrointestinal tumors and renal cancer. Somatic or germline mutation of genes encoding enzymes catalyzing pivotal steps of the TCA cycle not only disrupts cellular respiration, but also causes severe alterations in mitochondrial metabolite pools. These latter alterations lead to aberrant accumulation of “oncometabolites” that, in the end, may lead to deregulation of the metabolic adaptation of cells to hypoxia, inhibition of the DNA repair processes and overall pathological changes in gene expression. In this review, we will address the TCA cycle mutations leading to the development of PPGL, and we will discuss the relevance of these mutations for the transformation of neural crest-derived cells and potential therapeutic approaches based on the emerging knowledge of underlying molecular alterations.

## 1. Metabolism and Cancer

Almost a century ago, Nobel Prize winner Otto Warburg described how cancer cells can reprogram glucose metabolism by dramatically increasing the rate of glucose uptake, which is fermented to produce lactate even in the presence of oxygen and fully functioning mitochondria [1]. This observation suggested that defects in mitochondrial respiration could be the underlying cause of cancer. Although this aerobic glycolytic mechanism, known as the Warburg effect, has been studied extensively, its benefits for cell growth and survival are not well understood. In fact, nowadays it is more accepted that genetic events occurring in cancer cells are the cause of the alterations in metabolism observed by Warburg in the 1920s. At the beginning of the 21st century, the first mutations in the *SDHD* gene were reported, providing for the first time a link between germline alterations in a metabolic gene and the development of cancer, and demonstrating how disruption of mitochondrial respiration may lead to tumor development [2]. Moreover, the description of the first germline mutations in the *SDHD* gene in patients with hereditary pheochromocytoma (PCC) and paraganglioma (PGL) (together referred to as PPGL) marked a milestone in the study of this rare disease. 

## 2. Germline or Somatic Disruption of the Tricarboxylic Acid (TCA) Cycle Leads to PPGL Development

**Figure 1:**
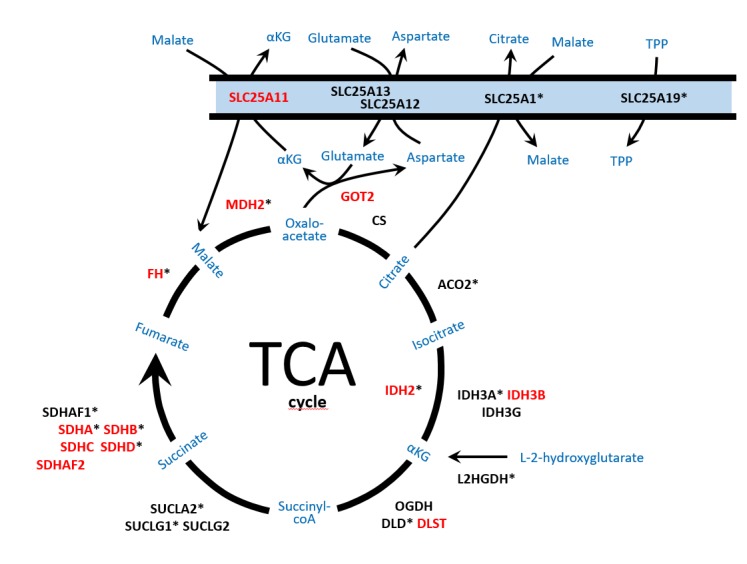
Schematic representation of the enzymes and mitochondrial metabolic pathways, tricarboxylic acid (TCA) cycle, malate/aspartate shuttle, nicotinamide adenine dinucleotide (NADH) exchange and metabolite efflux from the mitochondria, involved in pheochromocytoma and paraganglioma (PPGL) development and/or neurodegenerative disorders. Enzymes reported as altered in PPGL are denoted in red capital letters. Enzymes involved in neurodegenerative disorders are denoted with an asterisk. SLC25A11: solute carrier family 25 member 11; SLC25A12/SLC25A13: carriers solute carrier family 25 members 12/13; SLC25A1: solute carrier family 25 member 1; SLC25A19: solute carrier family 25 member 19; GOT2: mitochondrial glutamic-oxaloacetic transaminase 2; FH: fumarate hydratase; MDH2: mitochondrial malate dehydrogenase; CS: citrate synthase; ACO2: mitochondrial aconitase; IDH2: isocitrate dehydrogenase 2; IDH3A/IDH3B/IDH3G: subunits of isocitrate dehydrogenase 3; L2HGDH: L-2-hydroxyglutarate dehydrogenase; OGDH/DLD/DLST: subunits of the αKG (alpha-ketoglutarate) dehydrogenase complex; SUCLA2/SUCLG1/SUCLG2: subunits of succinyl-CoA synthetase; SDHA/B/C/D: subunits of the succinate dehydrogenase complex; SDHAF1/SDHAF2: succinate dehydrogenase assembly factors; αKG: α-ketoglutarate; TPP: thiamine pyrophosphate.

For a long time it was thought that the tricarboxylic acid (TCA) cycle was so crucial to the metabolism of living cells that any significant defect, including mutations affecting the pivotal enzymatic activities, would be highly unlikely and probably incompatible with life. To date, thirteen TCA cycle-related genes have been described to be involved in the development of different cancers such as cutaneous and uterine leiomyomas, gastrointestinal tumors, gliomas, renal cancer, and especially PPGL. Thus, ~23% of PPGLs are found carrying mutations in genes encoding energy metabolism enzymes such as the succinate dehydrogenase (SDH) subunits (SDHx genes), fumarate hydratase or fumarase (*FH*), malate dehydrogenase 2 (*MDH2*), isocitrate dehydrogenases 1 (cytosolic), 2 and 3 (*IDH1/2/3*), glutamic-oxaloacetic transaminase 2 (*GOT2*) and solute carrier family 25 member 11 (*SLC25A11*) (Figure 1).

PPGLs are neuroendocrine tumors derived from chromaffin cells of the adrenal medulla and from neural crest progenitors of extra-adrenal paraganglia. Sympathetic PPGLs, including PCC and thoracic-abdominal-pelvic (TAP) PGLs, are mostly catecholamine-producing tumors, whereas those derived from parasympathetic paraganglia, mainly located in the head and neck (H&N) region, are non-secreting tumors. Although PPGLs are predominantly benign and patients can be cured by surgical removal of these tumors, they present a significant morbidity and mortality due to the clinical aggressiveness of metastatic tumors (especially those carrying mutations in TCA cycle-related genes), for which therapeutic options remain scarce. To note, PPGL is considered a very rare disease with an incidence of 2–8 patients per million per year (1000–2000 new cases diagnosed worldwide every year). Therefore, the discovery of new susceptibility genes involved in hereditary predisposition to develop metastatic PPGLs, which is crucial for genetic counseling, guiding follow-up and developing targeted therapies, has only been possible through the joint efforts of many groups studying this extremely rare disease.

## 3. SDH Genes and PPGL

Germline loss-of-function mutations in *SDHA*, *SDHB*, *SDHC*, *SDHD* and *SDHAF2* (together accounting for 20% of all PPGLs) cause the well-characterized familial PGL syndromes known as PGL5 [3], PGL4 [4], PGL3 [5], PGL1 [2] and PGL2 [6], respectively (Table 1). Additional solid tumors such as gastrointestinal stromal tumors (GISTs) [7], clear cell renal cell carcinomas (ccRCCs) [8] and pituitary adenomas (PAs) [9] have been associated, albeit rarely, with these familial PGL syndromes [10].

**Figure 2:**
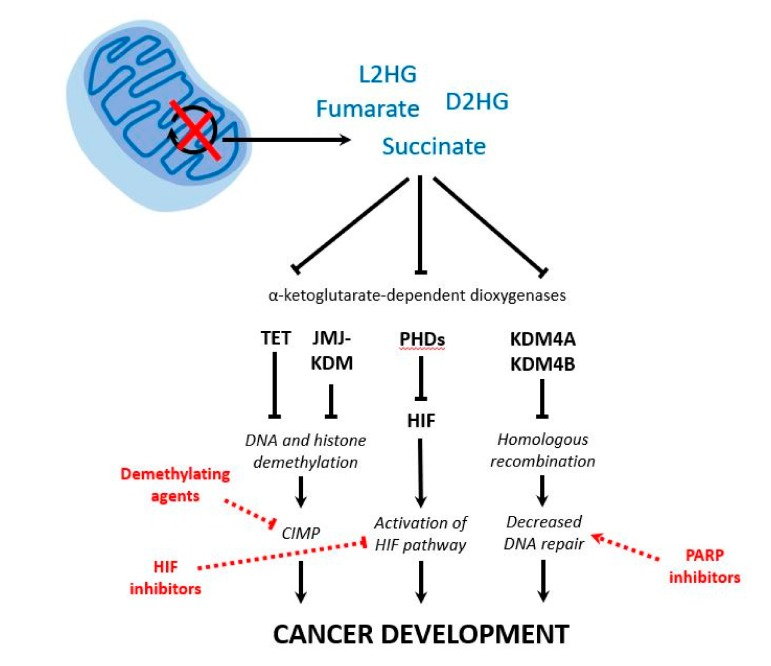
Schematic representation of the consequences of tricarboxylic acid (TCA) cycle disruption in pheochromocytomas and paragangliomas (PPGL). Upon disruption of the activity of pivotal TCA cycle enzymes, there is an accumulation of metabolites (i.e., succinate, fumarate, D2HG and L2HG). Their efflux from the mitochondria to the cytosol and their subsequent competition with α-ketoglutarate lead to the inhibition of α-ketoglutarate-dependent dioxygenases involved in DNA and histone demethylation, regulation of HIF, and homologous recombination. As a result, different mechanisms are proposed as the cause of tumorigenesis in PPGL: aberrant global hypermethylation (CIMP), activation of the HIF pathway and decreased DNA repair. Finally, different therapeutic options may target each altered pathway: demethylating agents, HIF inhibitors, and poly-(ADP-ribose)-polymerase (PARP) inhibitors, respectively. D2HG: D-2-hydroxyglutarate; L2HG: L-2-hydroxyglutarate; TET: ten-eleven translocation DNA hydroxylase; JMJ: Jumonji; KDM: histone lysine demethylase; PHDs: prolyl hydroxylase domain-containing proteins; HIF: hypoxia-inducible factor; CIMP: CpG island methylation phenotype.

The SDH enzyme complex is a hetero-oligomer that comprises four structural subunits: two hydrophilic catalytic subunits, SDHA and SDHB, and two hydrophobic subunits that anchor the catalytic ones to the inner mitochondrial membrane, SDHC and SDHD. The fifth SDH gene associated with PPGL development, *SDHAF2*, encodes a cofactor responsible for flavination of SDHA. The SDH complex catalyzes the oxidative dehydrogenation of succinate to fumarate in the TCA cycle [11], and it is also functionally involved in the electron transport chain forming the mitochondrial complex II. Germline or somatic mutations in any of the SDH genes (SDHx) cause disassembly of the mitochondrial complex, with loss of SDH enzymatic activity and thus triggering the accumulation of its substrate, succinate. When succinate accumulates pathologically, it acts as a competitor of alpha-ketoglutarate (αKG) to broadly inhibit the activity of αKG-dependent dioxygenases, such as ten-eleven translocation (TET) DNA hydroxylases and Jumonji (JMJ) histone lysine demethylases (KDM) [12,13]. This causes a global hypermethylation with a characteristic CpG island methylation phenotype (CIMP) profile in the tumors, which leads to altered gene expression and contributes to tumorigenesis; this same mechanism was earlier observed in glioblastomas [14] and ccRCCs [15] carrying metabolic alterations such as *IDH1/2* and *FH/SDHB* mutations, respectively. Apart from the aforementioned CIMP profile, the accumulation of succinate competitively inhibits the family of prolyl hydroxylase domain-containing proteins (PHD1-3), leading to hypoxia-inducible factor 1 α (HIF-1α) stabilization under normoxic conditions, and contributing to activation of the pseudohypoxic pathway [16,17]. More recently, it was reported that the succinate-mediated inhibition of two αKG-dependent dioxygenases, histone lysine demethylases KDM4A and KDM4B, leads to suppression of homologous recombination [18]. Moreover, the accumulation of succinate also causes downregulation of the enzyme responsible for the conversion of norepinephrine to epinephrine, thus inducing the characteristic noradrenergic phenotype of SDHx tumors [13]. All these processes orchestrated by succinate (and fumarate) accumulation (both referred to as oncometabolites) have been proposed to be involved in tumorigenesis (Figure 2) and/or in the particular phenotype of PPGLs carrying TCA cycle-related mutations. This will be discussed in more detail below. 

### 3.1. SDHD

Baysal and colleagues described in 2000 the first gene responsible for hereditary PGL [2]. *SDHD*, referred to at that time as *PGL1* (the gene responsible for hereditary PGL type 1), had been mapped before to chromosome band 11q23, and was identified by direct sequencing of the best candidate gene contained in a subsequently narrowed critical interval [19]. The initial link with head and neck (H&N) PGLs, highly vascular tumors mainly arising in the main sensor of blood oxygenation (the carotid body), was rapidly extended to PCCs and TAP PGLs [20,21,22]. Although the original reports proposed that maternal imprinting accounted for the inheritance pattern observed in *SDHD*-related pedigrees, recent studies found that the “almost exclusive” paternal transmission of the disease can be explained by a somatic genetic mechanism targeting both the *SDHD* locus and a paternally imprinted gene on 11p15.5 [23]. Regardless the mechanism involved, the preferential paternal transmission of the disease may lead to generation skipping, making genetic counselling challenging. Moreover, though it can be considered as a rare scenario, development of PPGL may occur after maternal transmission of an *SDHD* mutation [24,25,26]. The estimated risk of PPGL in *SDHD* mutation carriers (excluding probands) at age 60 years is 43.2% [11]. Regarding the clinical presentation of *SDHD* mutations carriers, they primarily develop (multiple) H&N PGLs (84% of cases), although up to 22% also develop TAP PGLs and 12–24% develop PCCs (mainly unilateral); 3–10% of carriers develop metastases [27,28,29]. GISTs, PAs and, more rarely, ccRCCs can develop in *SDHD* mutation carriers [30]. It seems that the type of mutation, either impairing or not SDHD stability, influences the mutation-associated phenotype [27].

### 3.2. SDHB

One year after the finding of the first *SDHD* germline mutations in patients with PGL, mutations in *SDHB*, the gene encoding the SDH iron-sulfur subunit, were described as the genetic cause of hereditary PGL type 4 [4]. Soon, the presence of either point mutations or gross deletions affecting *SDHB* far surpassed the prevalence of *SDHD* mutations in patients with PPGL. Overall, mutations in *SDHB* and *SDHD* account for the great majority (over 60%) of all SDHx-related PGL patients, and *SDHB* itself accounts for approximately 10% of all PPGLs [31]. Unlike *SDHD*-associated clinical manifestations (i.e., multiple H&N PGLs), *SDHB* mutation carriers are usually diagnosed with single tumors that can arise in different locations (i.e., PCC, TAP PGL and H&N PGL) [32]. Paradoxically, mutations in *SDHB* show one of the lowest penetrances amongst the SDHx genes (13–21% at the age of 50 years) [11,31,33,34,35], but patients carrying germline *SDHB* mutations present a higher risk of malignancy (~50%) than other SDHx mutation carriers [11,32,36,37]. This latter observation favors the recommendation of prioritizing genetic testing of this gene in patients affected with PPGL [38]. In fact, the presence of mutations in *SDHB* is the best biomarker of poor prognosis and malignancy in PGL syndromes [36,39]. In addition, the absence of SDHB immunostaining in tumor cells, probably caused by altered assembly or SDH complex stability, is a reliable identifier of PPGLs caused not only by *SDHB* mutations, but also by any other SDHx mutation [40]. Although there is no explanation yet, it has been reported that male *SDHB* mutation carriers are at higher risk of disease than females [31]. Amongst the five SDHx genes, *SDHB* is the one with the most hereditary extra-paraganglial manifestations, such as ccRCCs [8,41], oncocytomas [42,43], GISTs [7], and PAs [44].

### 3.3. SDHC

The SDHC subunit anchors, along with SDHD, the SDH complex to the mitochondrial inner membrane. Though *SDHC* was the second SDHx gene identified as a cause of hereditary PGL (PGL type 3) [5], the frequency of patients carrying mutations in this gene is much lower than *SDHB*- and *SDHD*-related PPGLs, accounting for less than 1% of the patients. *SDHC* mutations result primarily in benign and non-functional H&N PGLs, but they have also been identified in patients with sympathetic PGLs [45,46]. Although no somatic point mutation affecting *SDHC* has been reported to date in PPGL, postzygotic epimutations in the gene promoter region have been identified in patients with PGLs [47], Carney triad (GIST, pulmonary chondroma and PGLs) [48], and Carney-Stratakis syndrome (PGL and GIST) [49]. This specific molecular mechanism of inactivation of the *SDHC* gene was first described in GISTs [50], and has been found in patients developing more than one PGL and/or having syndromic features resembling a hereditary case, as occurs with endothelial PAS domain protein 1 (*EPAS1*, also known as *HIF2A*) and *H3F3A* postzygotic somatic mutations. To identify these “genetically hidden” SDHx cases, a negative immunohistochemistry for SDHB appears to be essential. Although until now there are few patients reported, it seems that in general *SDHC* mutation carriers have low risk of developing GISTs and PAs [11].

### 3.4. SDHA

Paradoxically, mutations affecting the gene encoding one of the two major catalytic subunits of the SDH complex were described subsequently to mutations affecting the other members of the complex. Homozygous germline mutations of *SDHA* in patients with Leigh syndrome have been known since 1995 [51], but the involvement of this gene in the development of PPGL was elusive until 2010 [3]. This can be explained because, although *SDHA* variants are significantly enriched in PPGL cases, several *SDHA* alleles show a high prevalence amongst the normal population (i.e., 0.1–1% in gnomAD), and were therefore not considered as causative mutations in PPGL patients [52]. Mutations in *SDHA* have the lowest penetrance of all major PGL predisposition genes (10% at age 70 years) [53], and therefore most *SDHA* mutation carriers will not manifest the disease. Familial PPGL related to mutations in *SDHA* shows a prevalence of 3%, especially in patients with PGL, although it is not rare to find mutations in cases developing PCC [54]. The presence of metastasis in *SDHA* mutation carriers is 12%, and extraparaganglial manifestations such as PAs, GIST and ccRCC have also been described [53,54,55].

### 3.5. SDHAF2

Only two different mutations in *SDHAF2*, encoding one of the known SDH complex assembly factors, have been described to date in families with H&N PGLs [6,56,57], and one of them (i.e., p.Gly78Arg) exhibits a founder effect in the Dutch population [58]. As occurs with *SDHD* mutation carriers, carriers of *SDHAF2* mutations show an exclusively paternal transmission of the disease, maybe because both genes are located on the same chromosome and may follow the same route to tumorigenesis [59]. Patients carrying mutations in *SDHAF2* develop PGLs only in the H&N region, and frequently (74% of mutation carriers) in multiple locations [60,61]. Though the *SDHAF1* gene has been found mutated in SDH-defective infantile leukoencephalopathy, no mutations have been reported in PPGL to date [62].

## 4. Other TCA Cycle-Related Genes

### 4.1. FH

Fumarate hydratase (FH) catalyzes the reversible hydration of fumarate to malate in the TCA cycle. Deficiency in FH activity leads to the accumulation of the oncometabolite fumarate and to the subsequent inhibition of multiple αKG-dependent enzymes, which drives critical epigenetic changes and signaling pathway activation. Thus, overabundance of fumarate, as occurs with succinate, leads to stabilization of HIF-1α, deregulation of DNA/histone methylation, increase of glutaminolysis and glycolysis, and production of reactive oxygen species (something not found in SDH-mutant PPGLs) [63]. All these altered phenomena could promote carcinogenesis by stimulating proliferation and cell survival. Inactivating germline mutations affecting the *FH* gene are the cause of hereditary leiomyomatosis and renal cell carcinoma (HLRCC) [64], but, as far as we know, no PPGL has been reported in families with HLRCC. In 2013, a germline mutation in *FH* was found in a patient with PCC by whole exome sequencing (WES) applied to a tumor displaying transcriptional and methylation (CIMP profile) similarities to SDH-mutant tumors [13]. Subsequently, the study of two large series of patients allowed the identification of additional patients carrying inactivating germline *FH* mutations [65,66]. The prevalence of alterations in the gene is about 1% in PPGL patients, and a metastatic phenotype and presence of multiple tumors located in the adrenal gland or in TAP paraganglia are the clinical characteristics of PPGLs associated with *FH* mutations. In addition, fumarate causes a non-enzymatic covalent modification of cysteine residues in proteins, S-(2-succinyl) cysteine (2SC) [67], that can be detected by immunohistochemistry and has been proposed as a biomarker for *FH*-associated neoplasia. This protein modification, called succination, is different from succinylation, another post-translational modification that will be discussed later in this review. The extent of succination and the implications it may have for the function of targeted proteins is poorly studied, but it seems that this process is more likely to lead to inactivation of critical enzymes [68,69].

### 4.2. MDH2

*MDH2* encodes the enzyme malate dehydrogenase 2, which is essential for the reversible oxidation of malate to oxaloacetate in the TCA cycle. This tumor suppressor gene was first reported mutated, with an incomplete penetrance, in a single family with multiple malignant PGLs [70]. Very recently, additional variants have been reported, accounting for <1% of the patients, and their involvement in noradrenergic PPGLs with malignant behavior has been suggested [71]. Tumors carrying *MDH2* mutations showed no accumulation of malate, and though knockdown of *MDH2* in HeLa cells triggered the accumulation of both malate and fumarate, the connection between mutations in this gene and tumorigenesis is not clear. Subsequent to the finding of *MDH2* mutations in PPGL, biallelic mutations of the gene were found as the cause of severe encephalopathy in pediatric patients [72], reinforcing the pathological role of alterations in this particular gene.

### 4.3. IDH Genes

Isocitrate dehydrogenases IDH1 (cytoplasmic) and IDH2 (mitochondrial) catalyze the oxidative decarboxylation of isocitrate to αKG. Recurrent, and mutually exclusive, mutations in the genes *IDH1* (involving R132) and *IDH2* (involving R172), result in neomorphic production of the oncometabolite D-2-hydroxyglutarate (D2HG) that ultimately causes the characteristic CIMP profile previously mentioned for succinate and fumarate accumulation [73]. Somatic mutations in the *IDH1* gene (p.R132C), frequently found in central nervous system tumors [74], have been rarely identified in PGLs (i.e., three benign tumors in TAP or H&N locations) [49,75,76]. On the other hand, only one somatic *IDH2* mutation has been found in a patient with a single H&N PGL by metabolome-guided genomic analysis [77]. Moreover, we recently found a germline truncating mutation affecting *IDH3B* in a patient with a single H&N PGL showing an altered αKG/isocitrate ratio and a CIMP-like profile [49]. Homozygous loss-of-function mutations in *IDH3B* have been found in families with retinitis pigmentosa, a hereditary neurodegeneration of rod and cone photoreceptors in the retina [78]. IDH3 is a heterotetramer, with two catalytic subunits encoded by *IDH3A* and two regulatory subunits encoded by *IDH3B* and *IDH3G*, which catalyzes the irreversible conversion of isocitrate to αKG in the TCA cycle. Although the finding of an *IDH3B* loss-of-function variant in a neuroendocrine tumor such as PGL suggests a causative role for this gene in the disease, and also for the other two genes encoding the remaining subunits of the tetramer, further studies are needed to definitively confirm this association.

### 4.4. SLC25A11 and GOT2

Another WES study applied to a tumor exhibiting an SDHx-like molecular phenotype (i.e., pseudohypoxic and CIMP profiles) in the absence of SDHx or *FH* mutations, identified a germline mutation in the *SLC25A11* gene, which encodes the mitochondrial α-KG/malate carrier [79]. Five additional patients, most of them developing metastatic TAP PGLs, were found in this study carrying *SLC25A11* mutations. This gene is not directly involved in the TCA cycle, but it participates in the exchange between two major intermediates of the cycle, which suggests that inactivation of other genes causing alterations in mitochondrial homeostasis can be responsible for PPGL development as well. Moreover, a single gain-of-function mutation in the *GOT2* gene, encoding the mitochondrial glutamic-oxaloacetic transaminase and also involved in stimulating the malate/aspartate shuttle, was recently reported in a patient with multiple metastatic PGL [49], further reinforcing the link between dysfunction of proteins involved in the exchange of metabolites between the mitochondria and the cytoplasm and PPGL.

### 4.5. New TCA Cycle-Related Genes Involved in PPGL Development

Recently, a recurrent germline variant affecting *DLST* was found in PPGL patients with multiple tumors [80]. DLST (dihydrolipoamide *S*-succinyltransferase) is one of the three components (the E2 component) of the 2-oxoglutarate dehydrogenase (OGDH) complex that catalyzes the overall conversion of αKG to succinyl-CoA and CO_2_. Accumulation of L-2-hydroxyglutarate (L2HG) was found both in *DLST*-mutated tumors and in DLST-knockout (KO) cells transfected with the mutated protein. Surprisingly, and despite the mentioned accumulation, *DLST*-mutated tumors did not exhibit a CIMP profile, but they showed methylation and expression profiles similar to those observed for *EPAS1*-mutated PPGLs, suggesting a link between DLST disruption and pseudohypoxia. Moreover, a high *HIF3A* expression and a positive DLST immunostaining exclusively found in tumors carrying TCA cycle mutations or *EPAS1* mutations further supported this pseudohypoxic link.

## 5. Metabolic Remodeling not Associated with TCA-Cycle Alterations

Apart from the mentioned TCA cycle alterations, metabolic reprograming of cancer cells can be achieved in PPGLs by other molecular mechanisms. Mutations in *EPAS1*, as well as the stabilization of HIF-1α occurring in *VHL*- and PHD-mutated PPGLs, trigger a pseudohypoxic switch of metabolism from mitochondrial respiration to glycolysis irrespective of oxygen levels. On the other hand, MYC deregulation caused by mutations in *MAX* may increase, in cooperation with HIF-2α, glucose uptake and glycolysis. Moreover, activating alterations of the phosphatidylinositol 3-kinase (PI3K)/AKT serine-threonine kinase (Akt)/mammalian target of rapamycin (mTOR) pathway (by loss-of-function mutations in *NF1* and *TMEM127*, or gain-of-function mutations in *RET*, *FGFR1* and *HRAS*) can also increase glycolysis through the transcription of glycolytic enzymes [63].

## 6. Inborn TCA Cycle Alterations: Neurodegenerative Disorders versus Cancer

The relevance of hereditary alterations in TCA cycle-related genes to the etiology of severe mitochondrial disorders is well known. Thus, autosomal-recessive mutations in almost any of the genes encoding the main enzymes of the TCA cycle lead to different forms of encephalopathies (Figure 1). This is the case for recessive germline mutations in *SUCLG1* [81] and *SUCLA2* [82]. Moreover, *ACO2* alterations cause infantile cerebellar-retinal degeneration [83] and severe optic atrophy and spastic paraplegia [84]; *IDH3A* mutations lead to severe encephalopathy in infancy [85]; mutations in *DLD* cause severe encephalopathy and hyperlactatemia with neonatal onset [86]; homozygous *IDH2* mutations provoke developmental delay, epilepsy, hypotonia, cardiomyopathy, and dysmorphic features [87]; *SDHAF1* mutations lead to infantile leukoencephalopathy [62]; *SDHA* alteration is a well-known cause of Leigh syndrome, cardiomyopathy and leukodystrophy [51]; recessive *SDHB* mutations lead to hypotonia and leukodystrophy [88] and *SDHD* mutations cause encephalomyopathy [89]; *FH* alterations cause progressive encephalopathy in early childhood [90]; and *MDH2* mutations provoke early-onset severe encephalopathy [72]. In addition, homozygous mutations in *SLC25A1*, encoding a mitochondrial citrate carrier, cause combined D-2- and L-2-hydroxyglutaric aciduria, severe neonatal epileptic encephalopathy, absence of developmental progress, and often early death [91], and mutations in *SLC25A19*, encoding a mitochondrial transporter of a TCA cycle cofactor (thiamine pyrophosphate), cause encephalopathy and progressive polyneuropathy [92]. Finally, *L2HGDH* alterations cause macrocephaly, developmental delay, epilepsy, and cerebellar ataxia [93].

Considering that complete abrogation of the TCA cycle in cells is highly unlikely to be compatible with cell viability, how is it possible that homozygous germline mutations in TCA cycle-related genes exist? The first mammalian model lacking a protein of the TCA cycle was obtained in 1997. Johnson et al. generated viable *DLD*^+/−^ mice, while the homozygous knockout animals (*DLD*^−/−^) died at early embryonic stages [94]. This also occurred with *SDHD* [95], *FH* [96], *DLST* [97], *SDHB* [13] and *SUCLA2*/*SUCLG2* [98] knockout mice. Overall, these data suggest that complete lack of activity of TCA cycle enzymes appears to be deleterious during embryonic development. These observations imply that homozygous or compound heterozygous variants in TCA cycle-related genes associated with neurological disorders do not completely abolish the corresponding enzymatic activity and retain part of their functionality. However, the presence of homozygous or compound heterozygous SDHx mutations in patients with neurological disorders gives rise to a controversial situation, which is the absence of tumors in mutation carriers. In heterozygous mutation carriers, this can be either accounted for by the low penetrance of TCA cycle-related mutations (especially in the case of *SDHA* and *SDHB* variants) or by the aforementioned modest effect of the alterations found in these patients. In addition, though clinical presentation and survival may be variable [99], patients carrying homozygous or compound heterozygous germline mutations in TCA cycle genes are usually diagnosed at a young age and die within a few years of diagnosis before reaching adulthood [100], so tumor diagnosis is unlikely to occur in these patients. However, taking into account that patients with L-2-hydroxyglutaric aciduria show increased risk of brain tumors [101], the tumorigenic potential of recessive mutations in TCA cycle-related genes cannot be ruled out. Both PPGL patients carrying TCA cycle-related mutations and patients with encephalopathies associated with the presence of recessive mutations in the aforementioned genes may benefit from therapeutic approaches targeting the aberrant DNA/histone methylation and the DNA-repair pathway, such as the use of demethylating agents (5-aza-2′-deoxycytidine or decitabine, for instance) or PARP inhibitors, respectively.

## 7. TCA Cycle-Related Omics Profiling in PPGLs

### 7.1. Pseudohypoxic Transcriptional Profile

The major regulator of cellular response to limited levels of oxygen (hypoxia) is the heterodimeric (composed of alpha and beta subunits) basic helix-loop-helix transcription factor HIF-1α. Under low oxygen conditions, HIF-1α (and also HIF-2α) activates the transcription of many genes involved in the metabolic adaptation to hypoxia, including transporters for increased glucose import (favoring anaerobic growth by glycolysis) and genes encoding angiogenesis factors [102]. The regulation of HIF-1α activity involves oxygen-limited hydroxylation of prolyl residues carried out by PHD1-3 prolyl hydroxylases. This prolyl hydroxylation induces the binding of HIF-1α to the von Hippel–Lindau (VHL) protein-associated complex, which is in charge of targeting HIF-1α by ubiquitination for proteosomal degradation [103,104]. Prolyl hydroxylation of HIF-1α requires oxygen, iron, and αKG, and the reaction produces succinate. Under hypoxia, prolyl hydroxylation is inhibited, HIF-1α is not ubiquitinated and this leads to the transcription of HIF-responsive genes. However, cancer cells may reproduce the hypoxic gene expression signature regardless of the oxygen condition in a process known as pseudohypoxia. This process is the hallmark of the VHL syndrome, in which mutations of *VHL* lead to HIF-1α stabilization in normoxia, leading to the development of tumors (e.g., ccRCCs and PPGLs). The pseudohypoxic transcriptional profile associated with the presence of mutations in TCA cycle-related genes in PPGL was first described by Patricia L Dahia in 2005. In this pioneering study, it was shown that SDH mutations, and the subsequent accumulation of succinate, caused a pseudohypoxic transcription profile similar to the one originated by VHL dysfunction [16,63,105]. Chronic pseudohypoxic signaling could be a mitogenic tumor initiator in neuroendocrine cells, and therefore inappropriate HIF-1α or HIF-2α persistence due to loss of SDH function in PPGL could drive tumorigenesis [106,107]. To note, the only environmental risk factor described in PPGL is chronic hypoxia which, in populations living at high altitude, leads to an increased incidence of H&N PGLs [108,109,110,111]. However, though the accumulation of oncometabolites is undoubtedly linked to tumor development in PPGLs, the subsequent pseudohypoxic response is not the only downstream mechanism proposed to explain tumorigenesis caused by TCA cycle dysfunction. The identification and validation of HIF-2α as one of the main oncogenic drivers in PPGLs [112,113,114] provides a rationale for exploring direct inhibition of HIF-2α as a therapeutic target in metastatic patients [115]. Preclinical studies have shown that HIF-2α antagonists are capable of inhibiting tumor growth in several models of renal cancer, and clinical trials of the first-in-class HIF-2α antagonist PT2385 [(S)-3-((2,2-difluoro-1-hydroxy-7-(methylsulfonyl)-2,3-dihydro-1H-inden-4-yl)oxy)-5-fluorobenzonitrile] have achieved promising results in ccRCC [116]. These findings set the stage for future trials focused on metastatic PPGL, a tumor type that, similar to ccRCC, harbors the pseudo-hypoxia molecular signature as its main molecular hallmark [117,118].

### 7.2. TCA-Cycle Mutations and CpG Island Methylator Phenotype (CIMP)

The association between the presence of SDHx mutations and CIMP in PPGL has been known since 2008 [119]. Geli et al. performed a quantitative evaluation of promoter methylation of a set of tumor suppressor genes (i.e., *RASSF1A*, *RASSF5*, *CDKN2A, RARB*, *TNFRSF10D, CDH1*, and *APC*), and found that five of seven tumors exhibiting a targeted CIMP profile were mutated in *SDHB*. Moreover, this particular CIMP profile (defined by the presence of methylation in at least three of the genes included in the analysis) was associated with metastatic behavior and extra-adrenal location (both clinical characteristics related to SDHx mutations). Later, it was published that the accumulation of fumarate and succinate, upon *FH* or SDHx mutations respectively, led to enzymatic inhibition of multiple α-KG-dependent dioxygenases and consequent alterations of genome-wide histone and DNA methylation, linking the TCA cycle mutations to tumorigenesis by their effect on the epigenome [12]. Interestingly, and like a snake that bites its tail, the methylation of the promoter of *SDHC* is a very well-known mechanism leading to CIMP in GIST [50] and PPGLs [47,49]. In 2013, two separate studies based on DNA methylation profiling of samples carrying SDHx mutations further explored the connection between metabolic disruption and altered epigenetic modifications [13,120]. These studies uncovered that SDH deficiency, and the subsequent accumulation of succinate, led to DNA hypermethylation in multiple tumor lineages (e.g., PPGLs and GISTs). These epigenomic changes were particularly severe in *SDHB*-mutated tumors, potentially explaining their malignancy. To note, one of these methylation-based studies also uncovered mutations in *FH* as a cause of hereditary PPGL [13], expanding the CIMP to another TCA cycle-related gene beyond the SDHx genes. In addition, a sporadic PPGL has been also described showing CIMP associated with the presence of a *IDH1* mutation [49] but, as previously mentioned, tumors carrying *DLST* mutations do not exhibit the characteristic CIMP profile despite their accumulation of TCA metabolites [80]. To date, the presence of mutations in other genes involved in the epigenetic machinery in PPGLs exhibiting hypermethylation [121,122], and the finding of tumors with a CIMP profile not associated with mutations in any TCA cycle-related gene [49], further suggest that other genes and pathways may be involved in this particular phenotype. Additional studies are required to elucidate whether the accumulation of metabolites, and the subsequent CIMP, is the cause or a consequence of the tumorigenic process and the adverse outcome associated with some TCA cycle mutations in PPGL.

### 7.3. Metabolome-Guided Genetic Characterization of the TCA Cycle

Since a link between pathological accumulation of TCA cycle metabolites and tumorigenesis was established, the study of the PPGL-associated metabolome has been used in the characterization of tumors carrying common genetic alterations, and additionally, in the identification of new candidate genes [49], the interpretation of genetic variants, and the improvement of diagnostics [77,123,124]. Mass spectrometry-based measurements of succinate:fumarate ratios allow to distinguish between SDH-deficient PPGLs and tumors without dysfunction of the SDH complex [123]. This is true, except in the case of some H&N PGLs, which may exhibit higher fumarate levels and therefore lower succinate:fumarate ratios compared to those at adrenal or TAP locations. The absence of this particular metabolic signature in some H&N PGLs is probably due to their higher content of stromal cells that dilute the signal from the tumor cells. In addition, alterations in the metabolites’ ratios can be used to identify hidden alterations in other TCA cycle-related genes. Thus, a tumor with a gain-of-function *GOT2* mutation exhibited a high succinate:fumarate ratio, probably due to the accumulation of αKG and its subsequent conversion to succinate [49]. Moreover, high fumarate:malate ratios have been observed in PPGLs carrying *FH* mutations, high absolute values of D2HG can be detected in PPGLs carrying *IDH1/2* mutations [49,77], and accumulation of L2HG was found both in *DLST*-mutated PPGLs and in DLST-KO cells transfected with the mutated protein [80].

In addition, hypermethylation (and the subsequent downregulation) of genes involved in the biosynthesis (*PNMT*, *DRD2* and *SULT1A1*), transport (*SLC6A2*), and secretion (*NPY*) of catecholamines found in SDH- and FH-mutant PPGLs [13], may contribute to the immature catecholamine phenotypic features of PGLs carrying mutations in TCA cycle-related genes [124]. Thus, the reduced *PNMT* expression reported in SDH-mutant tumors has been proposed to cause the predominant secretion of noradrenaline or dopamine observed in these tumors.

## 8. Link Between Defective TCA Cycle and DNA Repair 

On the whole, mutations affecting TCA cycle-related genes are associated with metastatic PPGLs for which curative chances, if any, are very limited. For that reason, the recently reported link between accumulation of TCA cycle oncometabolites and homologous recombination paves the way for new therapeutic approaches to the treatment of metastatic PPGL. Moreover, FH enzymatic activity is required for the cellular DNA damage response to double-strand breaks, and it is known that it is involved in the non-homologous end joining repair pathway [125,126]. Thus, in this unexpected connection between metabolism and DNA repair, the excess of fumarate and succinate not only would inhibit αKG-dependent dioxygenase activities, specifically the lysine demethylases KDM4A and KDM4B, but also would suppress the homologous recombination pathway [18]. This blockage would avoid the maintenance of genomic integrity and would make cells vulnerable to synthetic-lethal targeting with poly [adenosine diphosphate (ADP)]-ribose polymerase (PARP) inhibitors. Recent findings have revealed that the expression of mitochondrial complex I core subunits were upregulated in PPGLs with a pseudohypoxic profile. This augmented complex I activity increases intracellular nicotinamide adenine dinucleotide (NAD^+^) levels, which serves as an important cofactor to support the PARP DNA repair pathway. Thus, pseudohypoxic PPGLs would present more efficient DNA repair, resulting in potential chemoresistance.Interestingly, a combined treatment with a PARP inhibitor and temozolomide improved cytotoxicity in vitro, reducing tumor proliferation and metastatic lesions with prolonged overall survival in mice with SDHB-KO allografts [127].

## 9. Defective TCA Cycle Metabolism, Succinylation of Histones and Transcriptional Responses

In addition to the mentioned effects that the accumulation of TCA cycle metabolites has on overall DNA methylation, there are other less known implications of disruption of the cycle. Lysine succinylation is a known post-translational protein modification [128] that when occurring in histones may affect chromosome structure and function [129]. It was recently demonstrated that a fraction of the OGDH complex localizes in the nucleus and binds to lysine acetyltransferase 2A (KAT2A) in the promoter regions of genes [130], and this nuclear translocation of the complex depends on a nuclear localization sequence in DLST. Preventing the OGDH complex from entering the nucleus avoids nuclear generation of succinyl-CoA and the subsequent KAT2A-dependent H3 succinylation, reducing gene expression and inhibiting tumor cell proliferation and tumor growth. These results are consistent with previous observations linking nucleosome succinylation with enhanced in vitro transcription. These interesting findings not only might explain the tumorigenic potential of DLST, as part of the OGDH complex, but also open a new avenue of research into the connection of metabolism and cancer. A subsequent study also demonstrated a potential role of chromatin succinylation in modulating gene expression using an inducible cell culture model of SDH loss, which results in accumulation of succinyl-CoA [131]. This study also demonstrated that defective TCA cycle metabolism results in a DNA repair defect. Chromatin succinylation may thus represent a mechanism by which metabolism modulates both genome-wide transcription and DNA repair activities.

Apart from the aforementioned effect on the succinylation of histones, inhibition of the OGDH complex also reduces lysine succinylation of cytosolic and mitochondrial proteins altering rates of enzymes and pathways, especially mitochondrial metabolic pathways [132,133]. Moreover, ablation of specific enzymes of the TCA cycle affects the availability of succinyl CoA and global enzymatic and non-enzymatic succinylation patterns [134], providing a novel mechanism in which mitochondrial intermediates act as sensors to regulate metabolism.

## 10. Can We Open New Therapeutic Avenues for TCA Cycle-Altered PPGLs?

Apart from the known approaches to treating malignant PPGLs [135,136], we will discuss here novel therapeutic strategies that should be pursued for tumors carrying alterations in the TCA cycle. Though overall PPGLs present a low degree of chromosome instability, about 2–3% of tumors show chromothripsis involving chromosome arm 1p, where SDHB is located [129]. Chromothripsis phenomenon is a form of genome instability, associated with poor prognosis [137,138], in which one or a few chromosomes are affected by an alternating copy number profile with both loss and retention of heterozygosity [139,140]. One of the mechanisms involved in the initiation of chromothripsis is telomere dysfunction that leads to attrition of chromosome ends [141]. Subsequently, cancer cells showing this phenomenon become stabilized in order to avoid additional chromosomal aberrations that would be incompatible with cell survival. A well-known telomere stabilization mechanism involves the presence of high levels of *TERT* mRNA, mainly through amplification, promoter point mutations, methylation or rearrangements. Interestingly, increased *TERT* expression and telomere length has been observed in chromothripsis-positive ependymomas and glioblastomas [142], and in high-stage neuroblastomas [143] among other cancers, suggesting that telomere maintenance pathways may represent therapeutic targets in chromothripsis-positive tumors. With regard to PPGL, high to moderate *TERT* expression has been observed in 18–51% of patients, which are often metastatic cases [75,144,145], and in an important proportion of patients associated with deficiency of SDHx [146]. Moreover, the presence of somatic *ATRX* mutations, found preferentially in *SDHB*/*FH*-mutated tumors, and the associated alternative lengthening of telomeres have been described as an independent risk factor for metastatic PPGL [75,146,147]. It is tempting to speculate that metastatic PPGLs related to mutations in other TCA cycle genes that also exhibit telomere dysfunction, could benefit from the multiple telomerase-targeting therapeutic strategies that have been pursued in the past two decades (reviewed in [148]). 

Another important avenue of potential treatments for metastatic PPGLs focuses on investigating whether a specific driver mutation could be associated with the tumor’s immune profile, and therefore with the potential efficacy of immunotherapy. In this regard, one of the main problems related to the potential lack of response is immune evasion, in which the tumor microenvironment (TME) plays a pivotal role [149]. The TME can be influenced by different conditions, but there are two in particular that gain importance when considering PPGLs with TCA-mutated genes: the hypoxic state, and an aberrant production of metabolites. Whether the PPGL immune subtypes are genotype-specific, or if there is an enrichment of any immune subtype among *SDHB*-mutated tumors or among those related to TCA genes with a higher metastatic risk is still to be addressed. Regardless these upcoming knowledge, it seems clear that a highly heterogeneous disease such as PPGL will benefit from a personalize treatment based on the specific genetic background, as well as from a deep characterization of the tumor microenvironment.

## 11. Conclusions

More than 20% of PPGLs are found carrying mutations in genes encoding TCA cycle metabolic enzymes. These mutations cause the disruption of the cycle, and the subsequent accumulation of “oncometabolites” lead to overall pathological changes in gene expression (i.e., by DNA methylation and post-translational protein modification), metabolic adaptation of cells to hypoxia, and DNA repair processes. Unraveling the pathological mechanisms associated with the presence of different PPGL mutations could pave the way to new personalized therapeutic approaches.

## Figures and Tables

**Table 1:** Summary of phenotypic and genetic features associated with the TCA cycle-related PPGL genes.

TCA Cycle Gene	Chr. Location	Mean Age	% of Germline Mutations (Penetrance by Age) ¥	Risk of Malignancy (%)	Predominant Tumor Location	Number of Tumors (% Multiple)	BC	Related Syndromes; Associated Tumors
*SDHD*	11q23.1	35y	9–10 (43%, 60y)	Low (3–10%)	H&N > TAP > PCC	M (56%)	NA, DA	PGL1, Carney-Stratakis syndrome, encephalomyopathy *; ccRCC, GIST, PA
*SDHB*	1p36.13	30y	10 (13–21%, 50y)	High (30–50%)	TAP > H&N > PCC	S>M (20–25%)	NA, DA	PGL4, Carney-Stratakis syndrome, hypotonia and leukodystrophy *; ccRCC, GIST, PA
*SDHC*	1q23.3	40–50y	1–5 (25%, 60y)	Low (<3%)	H&N > TAP > PCC	S>M (15–20%)	NA, DA	PGL3, Carney-Stratakis syndrome; GIST, PA
*SDHA*	5p15.33	40y	3 (10%, 70y)	Moderate (12%)	H&N > TAP >> PCC	S>M (10–15%)	NA	PGL5, Leigh syndrome *, cardiomyopathy*, leukodystrophy *; ccRCC, GIST, PA
*SDHAF2*	11q12.2	30–40y	0.1–1	Low	H&N >> PCC	M (74%)	NA	PGL2; infantile leukoencephalopathy *
*FH*	1q42.1	-	1	High (60%)	PCC + TAP> H&N	M (60%)	NA	HLRCC, progressive encephalopathy in early childhood *; multiple cutaneous and uterine leiomyomatosis; cutaneous and uterine leiomyomas, type 2 papillary renal carcinoma
*MDH2*	7q11.23	45y	<1	High (50%)	TA	S > M (33%)	NA	Early-onset severe encephalopathy *
*IDH1*	2q34	>60y	NA	Low	TAP, H&N	S	NA	
*SLC25A11*	17p13.3	59y	1	High (70%)	TAP >> H&N	S	NA	
*DLST*	14q24.3	29y	<1	Low	TAP >> PCC	M (100%)	NA	

Chr: chromosome; **¥**: penetrance data was included for genes with prevalence >1%; TCA: tricarboxylic acid; PGL: paraganglioma; NA: not applicable; H&N: head and neck paraganglioma; TAP: thoracic-abdominal-pelvic paraganglioma; PCC: pheochromocytoma; S: single; M: multiple BC: Biochemical predominant secretion; NA: noradrenergic (predominant secretion of noradrenaline/normetanephrine); A: adrenergic (predominant secretion of adrenaline/metanephrine); DA: dopaminergic (secretion of dopamine/3-methoxytyramine); GIST: gastrointestinal stromal tumor; ccRCC: clear cell renal cell carcinoma; PA: pituitary adenoma; HLRCC: hereditary leiomyomatosis and renal cell cancer. Other genes found mutated in single cases, such as *GOT2*, *IDH2* or *IDH3B*, are not included in the table. *: caused by autosomal-recessive mutations.
